# Dual versus monotherapy with bronchodilators in GOLD group B COPD patients according to baseline FEV_1_ level: a patient-level pooled analysis of phase-3 randomized clinical trials

**DOI:** 10.1186/s12931-021-01648-5

**Published:** 2021-02-12

**Authors:** Jieun Kang, Jae Seung Lee, Sei Won Lee, Jung Bok Lee, Yeon-Mok Oh

**Affiliations:** 1 Division of Pulmonary and Critical Care Medicine, Department of Internal Medicine, Ilsan Paik Hospital, Goyang-si, Gyeonggi-do Republic of Korea; 2 Department of Pulmonary and Critical Care Medicine, Asan Medical Center, University of Ulsan College of Medicine, 88 Olympic-ro 43-gil, Seoul, Republic of Korea; 3 Department of Clinical Epidemiology and Biostatistics, Asan Medical Center, 88 Olympic-ro 43-gil, Seoul, Republic of Korea

**Keywords:** Chronic obstructive pulmonary disease, FEV_1_, Dual therapy, Monotherapy

## Abstract

**Background:**

Which patients should receive dual therapy as initial treatment for chronic obstructive pulmonary disease (COPD) is only loosely defined. We evaluated if a lower forced expiratory volume in 1 s (FEV_1_) identifies a population more likely to benefit from dual therapy than monotherapy among group B COPD patients in whom Global initiative for Chronic Obstructive Pulmonary Disease (GOLD) recommends monotherapy as initial treatment.

**Methods:**

This was a patient-level pooled analysis of phase-3 randomized controlled trials involving dual bronchodilators. Study patients were classified into two groups based on the FEV_1_ of 50% of the predicted value (GOLD I/II versus GOLD III/IV). We evaluated the efficacy of dual versus monotherapy (long-acting beta-2 agonist [LABA] or long-acting muscarinic antagonist [LAMA]) between these two groups in the following outcomes: changes in trough FEV_1_, the St. George’s Respiratory Questionnaire (SGRQ) score, the proportion of SGRQ responders, time to first exacerbation, and risk of adverse events.

**Results:**

A total of 14,449 group B patients from 12 studies were divided into GOLD III/IV (n = 8043) or GOLD I/II group (n = 6406). In the GOLD III/IV group, dual therapy was significantly more effective in improving FEV_1_, reducing SGRQ scores, and achieving a higher proportion of SGRQ responders compared with either LABA or LAMA. Dual therapy also showed a significantly longer time to first exacerbation compared with LABA in the GOLD III/IV group. In contrast, in the GOLD I/II group, the benefits of dual therapy over monotherapy were less consistent. Although dual therapy resulted in significantly higher FEV_1_ than either LABA or LAMA, it did not show significant differences in the SGRQ score and proportion of SGRQ responders as compared with LABA. The time to first exacerbation was also not significantly different between dual therapy and either LABA or LAMA in the GOLD I/II group.

**Conclusions:**

Dual therapy demonstrated benefits over monotherapy more consistently in patients with lower FEV_1_ than those with higher FEV_1_.

## Background

Chronic obstructive pulmonary disease (COPD) is one of the major causes of chronic morbidity and mortality worldwide [1–3]. It is characterized by irreversible airflow limitation and respiratory symptoms such as cough, sputum, and dyspnea [4]. The severity of airflow limitation is represented by forced expiratory volume at 1 s (FEV_1_). Although the importance of FEV_1_ is acknowledged, it does not determine treatment according to the Global initiative for Chronic Obstructive Lung Disease (GOLD) document [4]. GOLD classifies COPD patients and guides initial treatments based on symptom severity and exacerbation frequency [5, 6].

GOLD group B represents symptomatic patients with a low risk of exacerbation. According to the GOLD recommendations, the initial treatment choice is a long-acting bronchodilator, either a long-acting beta-2 agonist (LABA) or long-acting muscarinic antagonist (LAMA), with no preference between the two [6]. Dual therapy with LABA/LAMA is recommended as a step-up option [6]. Given that LABA and LAMA have different mechanisms of action [7, 8], dual therapy may provide greater benefits in terms of lung function improvement and symptom relief [9–11]. However, GOLD recommends dual therapy as an initial treatment only in patients with severe symptoms [6].

Considering that GOLD group B includes heterogeneous patients with a wide range of FEV_1_, some patients may not have a sufficient treatment response from LABA or LAMA alone. In particular, patients with a higher degree of airflow limitation at baseline may benefit from treatment more intensive than monotherapy. However, there has been no evaluation of whether the magnitude of treatment difference between dual and monotherapy varies according to the baseline FEV_1_ in group B patients. This study compared the effects of dual therapy and monotherapy in group B COPD patients according to FEV_1_ level (50% of the predicted value [%pred.]) to test the hypothesis that the population with a lower FEV_1_ level (GOLD grades III/IV) is more likely to benefit from dual therapy.

## Methods

### Data sources

This study was a patient-level pooled analysis of phase-3 randomized controlled trials that evaluated the efficacy of a dual bronchodilator (LABA/LAMA) compared with either LABA or LAMA monotherapy. We obtained individual patient-level data from available trials provided by the sponsor companies. The dual bronchodilators of interest included glycopyrronium/indacaterol (Ultibro Breezehaler^®^, Novartis), umeclidinium/vilanterol (Anoro Ellipta^®^, GSK), and tiotropium/olodaterol (Spiolto Respimat^®^, Boehringer-Ingelheim). The study protocol was approved by the Institutional Review Board of Asan Medical Center (IRB No.: 2018-0298). The requirement for informed consent was waived due to the retrospective nature of the study.

Individual patient-level clinical trial data are available to outside researchers through ClinicalStudyDataRequest.com (CSDR). CSDR is a consortium of global pharmaceutical companies including, GlaxoSmithKline, Astellas Pharma, Bayer, Novartis, Roche, and Boehringer-Ingelheim, as well as academic research funders, including the Bill & Melinda Gates Foundation, the UK Medical Research Council, and The Wellcome Trust [12]. It was launched in 2013 to facilitate data sharing among independent investigators [12] by providing deidentified raw global clinical trial data from multiple sponsors [13]. Our study proposal was submitted via a web-based portal to the CSDR’s Independent Review Panel for a review of scientific importance and qualification of the research team. Data was analyzed in a closed system provided by CSDR with in-built statistical software. Data outside of CSDR could not be merged with data provided by CSDR.

### Eligibility criteria

We requested data from phase-3 randomized controlled trials that evaluated the efficacy of a dual bronchodilator (glycopyrronium/indacaterol, umeclidinium/vilanterol, or tiotropium/olodaterol) in COPD patients. The research had to meet the following criteria: (1) compare dual and single bronchodilators with/without a placebo arm, (2) a parallel design, (3) duration of longer than eight weeks, and (4) outcomes include changes in trough FEV_1_, St. George's Respiratory Questionnaire (SGRQ) total score, risk of acute exacerbation, or adverse events. Studies were excluded if: (1) the comparator was not relevant to the study purpose (e.g., comparison between dual and inhaled corticosteroid (ICS)/LABA; (2) the dual bronchodilator was not given as a fixed-dose combination; (3) it was performed on patients who were not responsive to monotherapy; and (4) information regarding baseline symptom levels (modified Medical Research Council [mMRC] grade, COPD assessment test [CAT] or SGRQ score) and previous exacerbation were not adequately addressed. We submitted the study proposal to CSDR on March 15, 2018, and gained access to the requested data on January 2, 2019.

A total of 12 studies were included in this study (Additional file 1: Figure S1). The list of included studies and the types of study endpoints are described in Additional file 1: Table S1. Briefly, all studies measured trough FEV_1_ and the development of adverse events. Total scores of SGRQ and acute exacerbation were evaluated in 8 and 10 studies, respectively.

### Study patients

All of the studies shared common inclusion criteria. Patients were diagnosed with COPD by spirometry (post-bronchodilator FEV_1_/forced vital capacity < 0.7), aged 40 years or older, and had a smoking history of more than 10 pack-years [7, 10, 11, 14–18]. The baseline characteristics of each treatment arm (excluding the placebo arm) in the 12 studies are shown in Additional file 1: Table S2.

This study included patients who belonged to group B according to the 2017 GOLD classification criteria. To classify patients into one of the four groups (A, B, C, and D), we determined symptom severity at the time of inclusion and the number of exacerbations in the previous year. If the baseline CAT score or mMRC grade was not provided, a baseline SGRQ score of 25 was used as a cutoff value as suggested by GOLD [5]. Additional file 1: Table S3 shows which criteria (CAT score, mMRC grade, and SGRQ score) were used in each study. Exacerbation was defined as an acute worsening of the patient’s respiratory symptoms beyond normal day-to-day variations requiring a change in medication. According to the 2017 GOLD classification criteria, patients who experienced none or one exacerbation not requiring hospitalization during the previous year were included in the study.

### Study outcomes

We divided study patients into GOLD I/II group (FEV_1_ ≥ 50%pred.) and GOLD III/IV group (FEV_1_ < 50%pred.) based on their baseline FEV_1_ level. We evaluated whether the efficacy of dual versus monotherapy differs between these two groups on the following outcomes: change in the trough FEV_1_, change in the SGRQ total score, the proportion of SGRQ responders, time to first exacerbation, and the risk of adverse events. A change in the trough FEV_1_ was the difference between pre-dose FEV_1_ values at baseline and at the end of each study as shown in Additional file 1: Table S4. An SGRQ responder was defined as a patient who achieved a minimum clinically important difference (MCID) threshold of 4 points.

### Statistical analysis

A professional statistician (JB Lee) performed all statistical analyses. A generalized linear mixed model using stratified study-effects or random study-effects was used to sum up individual patient-level data. For continuous variables, such as a change in trough FEV_1_ and SGRQ score from baseline, a linear mixed model with random effects was applied with an adjusted multiple treatment comparison. The least-squares mean change from baseline values for each treatment group were reported with their associated standard errors and a 95% confidence interval (CI). For binary data, we used a generalized linear mixed model with Penalized Quasi-likelihood estimation. The odds ratio and 95% CI were estimated for the binary data. Cox’s proportional hazard model with random effects was used to summarize time to the first exacerbation. The hazard ratio (HR) and the corresponding 95% CI were then estimated. All p-values were two-tailed, and p-values < 0.05 were considered statistically significant. All analyses were performed using the Statistical Analysis System (SAS) statistical software package, version 9.4. SAS Institute Inc., Cary, NC, USA.

## Results

### Baseline characteristics of the study patients

We identified 20204 patients who received either dual or monotherapy of a long-acting bronchodilator from 12 studies. Among them, 14449 patients were classified as group B based on the 2017 GOLD classification criteria. Table 1 describes the baseline clinical characteristics of all group B patients and according to the baseline FEV_1_ level. The GOLD III/IV group accounted for 55.7% (n = 8043) of all study patients. Their mean age was 64.7 years and the mean FEV_1_ value was 35.1%pred. In the GOLD I/II group, the mean age and mean FEV_1_ values were 64.6 years and 54.6%pred., respectively. Among the patients who received dual therapy, the number of patients treated with tiotropium/olodaterol was the highest, followed by umeclidinium/vilanterol in both groups. Tiotropium was the most frequently administered bronchodilator monotherapy in both groups.

**Table 1:** Baseline characteristics of GOLD group B patients according to FEV_1_ level

	All	GOLD III/IV	GOLD I/II
Number of patients	14449	8043	6406
Age	64.7 ± 8.6	64.7 ± 8.4	64.6 ± 8.9
Male	9960 (68.9)	5821 (72.4)	4139 (64.6)
Current smoker	6384 (44.2	3455 (43.0)	2929 (45.7)
Smoking pack-years	46.2 ± 22.2	44.3 ± 22.2	48.6 ± 22.0
Body mass index	26.8 ± 5.8	26.3 ± 5.8	27.4 ± 5.8
FEV_1_, L	1.2 ± 0.5	1.0 ± 0.3	1.5 ± 0.4
FEV_1_, %pred	43.8 ± 13.5	35.1 ± 9.3	54.6 ± 9.2
No of exacerbation in the preceding year			
0	11,778 (81.5)	6471 (80.5)	5307 (82.8)
1	2671 (18.5)	1572 (19.5)	1099 (17.2)
Treatment			
LABA/LAMA			
Tiotropium/olodaterol	4528 (31.3)	2510 (31.2)	2018 (31.5)
Umeclidinium/vilanterol	1487 (10.3)	796 (9.9)	691 (10.8)
Glycopyrronium/indacaterol	791 (5.5)	492 (6.1)	299 (4.7)
LAMA			
Tiotropium	4869 (33.7)	2651 (33.0)	2218 (34.6)
Glycopyrronium	469 (3.2)	298 (3.7)	171 (2.7)
Umeclidinium	522 (3.6)	289 (3.6)	233 (3.6)
LABA			
Indacaterol	624 (4.3)	411 (5.1)	213 (3.3)
Olodaterol	658 (4.6)	331 (4.1)	327 (5.1)
Vilanterol	501 (3.5)	265 (3.3)	236 (3.7)

Data are presented as means ± SD or number (%)

*FEV*_*1*_ forced expiratory volume at 1 s, *GOLD* Global initiative for Chronic Obstructive Lung Disease, *LABA* long-acting beta-2 agonist, *LAMA* long-acting muscarinic antagonist; *%pred.* % of the predicted value

### ***Trough FEV***_***1***_

**Fig. 1:**
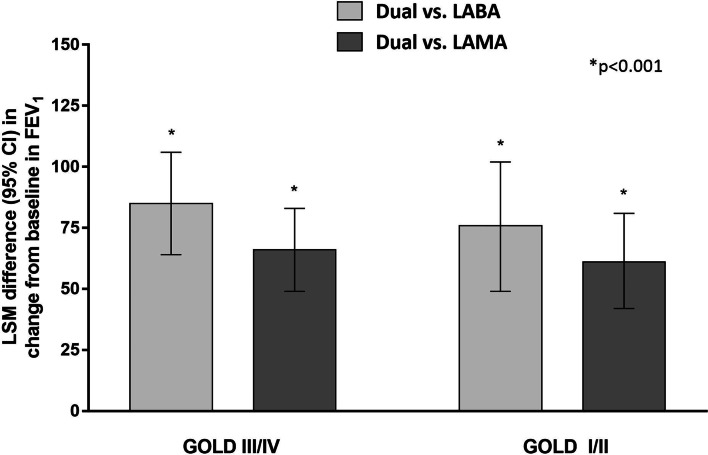
Difference between dual therapy vs. monotherapy in FEV_1_ change from baseline. *CI* confidence interval, *FEV*_*1*_ forced expiratory volume at 1 s, *GOLD* Global initiative for Chronic Obstructive Lung Disease, *LABA* long-acting beta-2 agonist, *LAMA* long-acting muscarinic antagonist, *LSM* least squares mean

In both the GOLD I/II and GOLD III/IV groups, dual therapy resulted in mean FEV_1_ improvement greater than MCID of 100 mL, whereas monotherapy did not (Table 2). The difference in the treatment effect between dual and monotherapy was statistically significant in both the GOLD I/II and GOLD III/IV groups, as shown in Fig. 1. Dual therapy provided a significantly greater FEV_1_ improvement than monotherapy, regardless of the comparator, LABA or LAMA (all p-values < 0.001).

**Table 2:** Changes in trough FEV_1_ from baseline following dual or monotherapy of LABA and/or LAMA

	Intervention	Mean change in trough FEV_1_ (SD), L	Treatment difference vs. dual (95% CI), L	p-value
GOLD III/IV	Dual	0.147 (0.209)		
	LABA	0.062 (0.208)	0.085 (0.064–0.106)	< 0.001
	LAMA	0.081 (0.209)	0.066 (0.049–0.083)	< 0.001
GOLD I/II	Dual	0.150 (0.234)		
	LABA	0.074 (0.232)	0.076 (0.049–0.102)	< 0.001
	LAMA	0.088 (0.239)	0.062 (0.042–0.081)	< 0.001

*CI* confidence interval, *FEV*_*1*_ forced expiratory volume at 1 s, *GOLD* Global initiative for Chronic Obstructive Lung Disease, *LABA* long-acting beta-2 agonist, *LAMA* long-acting muscarinic antagonist, *SD* standard deviation

### SGRQ total score

**Fig. 2:**
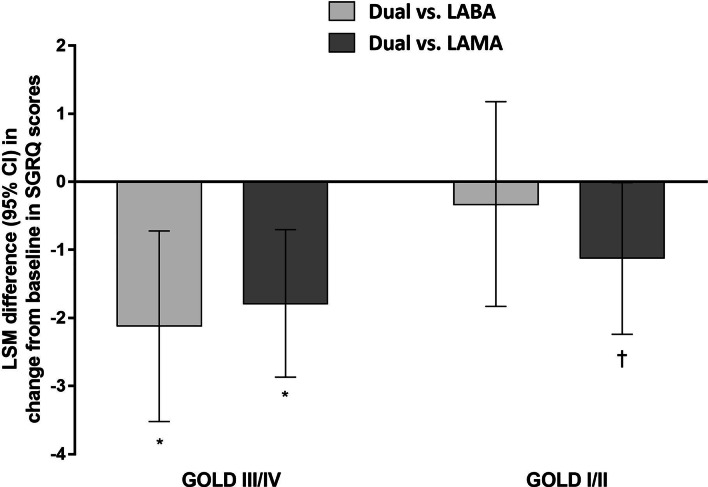
Difference between dual therapy vs. monotherapy in change from baseline in SGRQ score. *CI* confidence interval, *GOLD* Global initiative for Chronic Obstructive Lung Disease, *LABA* long-acting beta-2 agonist, *LAMA* long-acting muscarinic antagonist, *LSM* least squares mean, *SGRQ* St. George’s Respiratory Questionnaire

Table 3 shows the changes in the SGRQ total score following dual or monotherapy. Although dual therapy resulted in a greater reduction in the SGRQ score than monotherapy did, the difference between the two treatments had a different pattern in the GOLD I/II and GOLD III/IV groups. In the GOLD III/IV group, dual therapy reduced the SGRQ score by 7.63 points and was consistently, significantly greater than LABA (treatment difference, 2.12; p = 0.001) and LAMA monotherapy (treatment difference, 1.79; p < 0.001) as shown in Fig. 2. In the GOLD I/II group, however, dual therapy was significantly better than LAMA (treatment difference, 1.12; p = 0.048), but not LABA (treatment difference, 0.33; p = 0.868).

**Table 3:** Changes in total SGRQ score from baseline following dual or monotherapy of LABA and/or LAMA

	Intervention	Mean change in total score of SGRQ (SD)	Treatment difference vs. dual (95% CI)	p-value
GOLD III/IV	Dual	− 7.63 (12.09)		
	LABA	− 5.51 (12.76)	− 2.12 (− 3.52 to − 0.72)	0.001
	LAMA	− 5.85 (12.84)	− 1.79 (− 2.87 to − 0.70)	< 0.001
GOLD I/II	Dual	− 7.16 (12.49)		
	LABA	− 6.83 (12.54)	− 0.33 (− 1.83 to 1.18)	0.868
	LAMA	− 6.04 (12.89)	− 1.12 (− 2.24 to 0.01)	0.048

*CI* confidence interval, *GOLD* Global initiative for Chronic Obstructive Lung Disease, *LABA* long-acting beta-2 agonist, *LAMA* long-acting muscarinic antagonist, *SD* standard deviation, *SGRQ* St. George’s Respiratory Questionnaire

### SGRQ responders

**Fig. 3:**
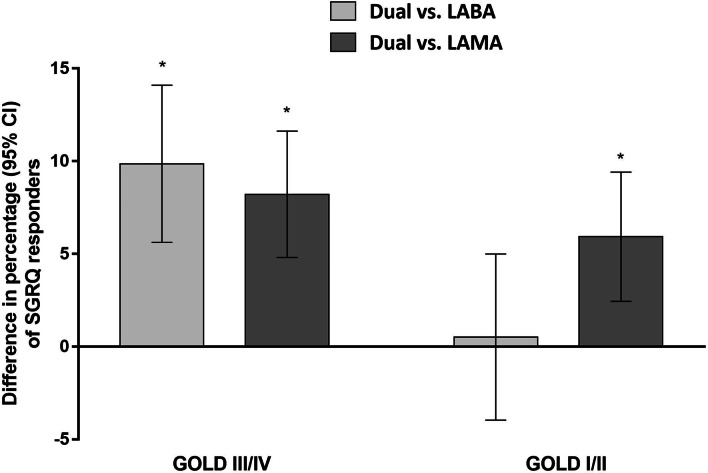
Difference between dual therapy vs. monotherapy in the percentage of SGRQ responders. *CI* confidence interval, *GOLD* Global initiative for Chronic Obstructive Lung Disease, *LABA* long-acting beta-2 agonist, *LAMA* long-acting muscarinic antagonist, *SGRQ* St. George’s Respiratory Questionnaire

Figure 3 shows the proportion of SGRQ responders in the GOLD I/II and GOLD III/IV groups. Similar to the changes in the SGRQ score, there was a different benefit pattern of dual therapy versus monotherapy, depending on the baseline FEV_1_ level. In the GOLD III/IV group, dual therapy resulted in a significantly higher proportion of SGRQ responders than both LABA and LAMA monotherapy. The treatment difference between dual vs. LABA and LAMA were 9.86% (95% CI 5.62–14.10; p < 0.001) and 8.21% (95% CI 4.81–11.62; p < 0.001), respectively. In the GOLD I/II group, dual therapy was significantly better than LAMA (treatment difference, 5.93%; 95% CI 2.44–9.41; p = 0.001), but not LABA monotherapy (treatment difference 0.52%, 95% CI − 3.96 to 4.99; p = 0.821).

### Time to first exacerbation

The mean exacerbation rates per 6 months are shown in Additional file 1: Table S5. Briefly, the exacerbation rates were 0.212, 0.188, 0.176 per 6 months in the LABA, LAMA, and dual therapy arm, respectively, in the GOLD III/IV group. In the GOLD I/II group, the rates were 0.139, 0.122, 0.108, in patients treated with LABA, LAMA, and dual therapy, respectively.

**Fig. 4:**
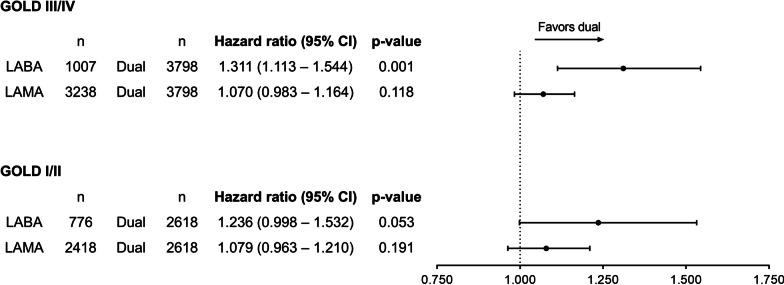
Difference between dual therapy vs. monotherapy in time to first exacerbation. *CI* confidence interval, *GOLD* Global initiative for Chronic Obstructive Lung Disease, *LABA* long-acting beta-2 agonist, *LAMA* long-acting muscarinic antagonist

Figure 4 shows the time to first exacerbation in patients who received dual therapy and those who received LABA or LAMA monotherapy. A different pattern was observed in the GOLD I/II and GOLD III/IV groups. In the GOLD III/IV group, dual therapy showed a significantly longer time to first exacerbation compared with LABA (HR, 1.311; 95% CI 1.113–1.544; p = 0.001), although there was no significant difference as compared with LAMA monotherapy. In the GOLD I/II group, dual therapy did not show any significant difference compared to both LABA and LAMA monotherapy.

### Adverse events

**Fig. 5:**
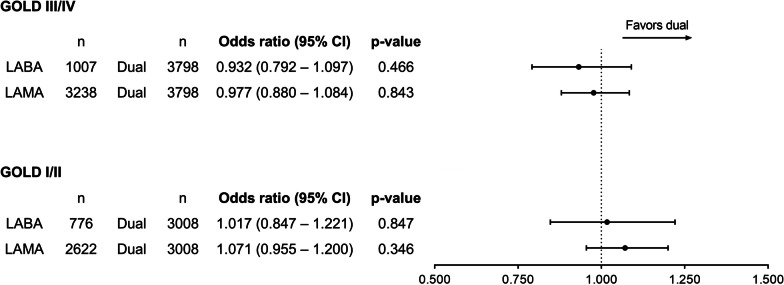
Difference between dual therapy vs. monotherapy in the risk of developing adverse events. *CI* confidence interval, *GOLD* Global initiative for Chronic Obstructive Lung Disease, *LABA* long-acting beta-2 agonist, *LAMA* long-acting muscarinic antagonist

There was no significant difference in the risk of any adverse events between dual and monotherapy in both the GOLD I/II and GOLD III/IV groups (Fig. 5).

## Discussion

This study compared the effects of dual therapy and monotherapy according to the baseline FEV_1_ level in GOLD group B patients using patient-level data from phase-3 randomized controlled trials. We found that the benefit of dual therapy over monotherapy was more prominent in the GOLD III/IV group. In the GOLD III/IV group, dual therapy was consistently more effective than LABA or LAMA in improving FEV_1_, reducing the SGRQ score, and achieving a higher proportion of SGRQ responders, and also resulted in a longer time to exacerbation compared with LABA. In contrast, in the GOLD I/II group, the benefit of dual therapy was less consistently shown in the study outcomes.

The purpose of COPD treatment is to reduce respiratory symptoms and prevent exacerbations [6]. Optimal treatment should be provided in order to achieve this goal. It remains undetermined if LABA or LAMA monotherapy is sufficient enough for group B COPD patients. In a previous study, the level of symptom burden was investigated in patients who were on maintenance treatment with LABA or LAMA alone [19]. Several domains of a respiratory questionnaire revealed greater impairment, including more dyspnea, in patients with FEV_1_ < 50%pred. than in those with ≥ 50%pred. Hence, there is a need to determine if patients with more severe airflow limitations require more intensive treatment than is currently recommended. In another study, patients who initiated maintenance therapy with a single bronchodilator showed a significantly shorter time to escalation to triple therapy (ICS/LABA/LAMA) than those who started treatment with a dual bronchodilator [20]. This may indirectly indicate a higher risk of additional treatment requirement in patients receiving monotherapy.

Given that the magnitude of FEV_1_ improvement was similar in the GOLD I/II and GOLD III/IV groups, it is interesting that the benefit of dual therapy over monotherapy regarding health-related quality of life appeared differently in the groups. An increase in FEV_1_ does not always indicate better results in patient-reported outcomes. For example, a previous study found that the proportion of SGRQ responders was not very different between indacaterol/glycopyrronium (63.7%) and indacaterol (63.0%), although indacaterol/glycopyrronium showed a significantly greater increase in trough FEV_1_ than indacaterol (1.45 L vs. 1.38 L; p < 0.001) [9]. One explanation for the difference shown in our study could be that patients who have less severe airflow limitations may not perceive the benefit of greater bronchodilation provided by dual therapy. Instead, in the GOLD I/II group, non-bronchodilator effects, such as enhanced mucociliary clearance, may have an important role in reducing SGRQ scores and preventing exacerbation [21]. As these effects are delivered by both dual therapy and monotherapy, the benefits of dual therapy over monotherapy might have appeared smaller in this group.

One may argue that the treatment difference in the SGRQ total score between dual therapy and monotherapy might not be large enough to be translated into a significant clinical benefit. However, the proportion of SGRQ responder was approximately 10% greater with dual therapy than either monotherapy in the GOLD III/IV patients. In the GOLD I/II group, dual therapy was significantly associated with a greater proportion of SGRQ responders than LAMA but the difference was smaller than that shown in the GOLD III/IV patients. These results indicate that patients with lower FEV_1_ are more likely to benefit from dual therapy than monotherapy in terms of health-related quality of life. Given that there was no significant difference in adverse events and dual therapy was associated with greater FEV_1_ improvement, the advantage of dual therapy in GOLD III/IV needs to be highlighted.

The time to first exacerbation appeared to be significantly longer with dual therapy than with LABA monotherapy in the GOLD III/IV group, although no difference was found when compared to LAMA monotherapy. In the GOLD I/II group, there was no significant difference in dual therapy vs. LABA or LAMA. Noninferiority of LAMA as compared with dual therapy in terms of exacerbation is consistent with the GOLD document, which recommends LAMA as an initial treatment for patients who frequently develop exacerbation [12, 18, 22]. However, for those who are symptomatic but have a low risk of exacerbation, the desired treatment goals may be an improved health-related quality of life and reduced symptoms rather than a reduced exacerbation. Therefore, the importance of dual therapy should not be underestimated in symptomatic patients. In line with this, dual therapy is indicated in patients with more impaired lung function (FEV_1_ less than 50%) according to the expert recommendation in the recently published Czech guidelines [23].

The population more likely to benefit from starting treatment with a dual bronchodilator has only been loosely defined [24]. In a previous study, Martinez et al. showed that patients with a higher symptom burden, represented as CAT score ≥ 20, are more likely to benefit from dual bronchodilator treatment than monotherapy. The treatment difference between dual and monotherapy in the SGRQ score changes and the use of rescue medication was greatest in those with a CAT score ≥ 20 [24]. Accordingly, GOLD reserves dual bronchodilator treatment for highly symptomatic patients [5]. However, it should be noted that patients with lower lung function may underestimate their symptom burden due to unconsciously restricted physical activity [25, 26]. This will potentially lead to undertreatment in patients with a high degree of airflow limitation. In contrast, FEV_1_ is an objective parameter that can be used simply in treatment decisions.

The proportion of patients with FEV_1_ < 50%pred. in group B is not negligible. There were 8043 patients with an FEV_1_ less than 50%pred., accounting for 55.7% of our study subjects. These patients would have been classified as group D if the 2011 GOLD classification criteria were applied. Since FEV_1_ is no longer considered in patient classification, a substantial proportion of patients have shifted from high-risk to low-risk groups [27–32]. Previously, Tudoric et al. analyzed data from a study of 3361 COPD patients in central and eastern Europe [27]. They reported that 20.4% of the entire cohort moved from group D to group B according to the revised classification system. In another study that retrospectively analyzed 1053 COPD patients, the proportion of group D decreased by more than half (from 34.2 to 11.6%), whereas group B increased from 40.6 to 63.2% [29]. In large COPD cohorts such as ECLIPSE, Copenhagen, and the COPD gene cohort, the proportion of patients classified as group D by FEV_1_ alone was higher than those classified by frequent exacerbation history [33, 34]. Given these findings, there may be a considerable number of patients in GOLD group B who can benefit from early dual therapy.

We should address the limitations of our study to better understand the results. First, we did not include studies that evaluated the efficacy of aclidinium/formoterol (Duaklir Genuair^®^, AstraZeneca), glycopyrronium/formoterol (Bevespi Aerosphere^®^, AstraZeneca), and other clinically available dual bronchodilators. The corresponding pharmaceutical company (AstraZeneca) was not part of CSDR, and their data were not available. Further, there is a concern that twice- and once-daily medications differ in efficacy [35]. In fact, a previous meta-analysis found less FEV_1_ improvement with aclidinium/formoterol than with glycopyrronium/indacaterol or umeclidinium/vilanterol [36]. Second, the study patients included those who received ICS. The study outcomes might have been affected by not only the bronchodilator treatment but also ICS. However, in a recent study performed in non-ICS users, dual therapy resulted in greater lung function improvement and a significant reduction in clinically important deterioration than monotherapy [37]. Third, only one study (DYNAGITO) evaluated the acute exacerbation risk as a primary outcome. Although 9 of the 12 studies provided data on the development of acute exacerbation, except for DYNAGITO, they were not powered for this outcome. Of note, DYNAGITO included the greatest number of patients, and the acute exacerbation results might have been derived largely from DYNAGITO. Fourth, several outcomes were assessed in this study, but the problem of multiplicity was not considered. Although there is relatively less need to adjust for multiplicity in post-hoc studies, the results should be interpreted with caution. Lastly, we did not assess the cost-effectiveness of dual therapy. However, several previous studies have found that dual bronchodilators are cost-effective in COPD patients [38–40]. Particularly in South Korea, the costs are almost similar between dual and single bronchodilators.

In conclusion, dual therapy showed more consistent benefits over monotherapy in the GOLD III/IV group than in the GOLD I/II group. Among group B COPD patients, those with lower FEV_1_ may benefit from more intensive treatment.

## Supplementary Information

**Additional file 1:** **Table S1.** List of included studies and their endpoints. **Table S2. **Baseline patient characteristics (excluding placebo arms) included in the 12 studies. **Table S3.** Criteria used to classify symptom severity in each study. **Table S4.** Changes in the trough FEV_1_ (L) according to the treatment arm in each study and treatment difference between dual and monotherapy. **Table S5.** Rates of acute exacerbation per 6 months in patients treated with dual or monotherapy of LABA and/or LAMA. **Figure S1.** Study flow diagram.

## Data Availability

The datasets used and/or analyzed during the current study are available from the corresponding author on reasonable request.

## Abbreviations

CAT: COPD assessment test
CI: Confidence interval
COPD: Chronic obstructive pulmonary disease
CSDR: Clinical Study Data Request
FEV_1_: Forced expiratory volume in 1 s
GOLD: Global initiative for Chronic Obstructive Lung Disease
HR: Hazard ratio
ICS: Inhaled corticosteroid
LABA: Long-acting beta-2 agonist
LAMA: Long-acting muscarinic antagonist
MCID: Minimum clinically important difference
mMRC: Modified Medical Research Council
SGRQ: St. George’s Respiratory Questionnaire
