# Supplementary material for: Dual versus monotherapy with bronchodilators in GOLD group B COPD patients according to baseline FEV1 level: a patient-level pooled analysis of phase-3 randomized clinical trials
Source: Respir Res. 2021 Feb 12;22:55. doi: 10.1186/s12931-021-01648-5 (PMC7881547; doi:10.1186/s12931-021-01648-5)
Supplement: Supplementary file 1 — Additional file 1: Table S1. List of included studies and their endpoints. Table S2. Baseline patient characteristics (excluding placebo arms) included in the 12 studies. Table S3. Criteria used to classify symptom severity in each study. Table S4. Changes in the trough FEV1 (L) according to the treatment arm in each study and treatment difference between dual and monotherapy. Table S5. Rates of acute exacerbation per 6 months in patients treated with dual or monotherapy of LABA and/or LAMA. Figure S1. Study flow diagram. [file 12931_2021_1648_MOESM1_ESM.docx]

**Additional File 1**

**Dual versus monotherapy with bronchodilators in GOLD group B COPD patients according to baseline FEV_1_ level: A patient-level pooled analysis of phase-3 randomized clinical trials**

Jieun Kang^1^, Jae Seung Lee^2^, Sei Won Lee^2^, Jung Bok Lee^3*^, and Yeon-Mok Oh^2*^

^1^Division of Pulmonary and Critical Care Medicine, Department of Internal Medicine, Ilsan Paik Hospital, Goyang-si, Gyeonggi-do, Republic of Korea

^2^Department of Pulmonary and Critical Care Medicine, Asan Medical Center, University of Ulsan College of Medicine, Seoul, Republic of Korea

^3^Department of Clinical Epidemiology and Biostatistics, Asan Medical Center, Seoul, Republic of Korea

*These authors contributed equally to this work.

Corresponding author: Yeon-Mok Oh

Department of Pulmonology and Critical Care Medicine, Asan Medical Center, University of Ulsan College of Medicine, 88 Olympic-ro 43-gil, Songpa-gu, Seoul, 05505, South Korea

Phone: 82-2-3010-3136

Fax: 82-2-3010-6968

E-mail: [yeonmok.oh@gmail.com](mailto:yeonmok.oh@gmail.com)

Co-corresponding author: Jung Bok Lee

Department of Clinical Epidemiology and Biostatistics, Asan Medical Center, Seoul, Republic of Korea

Phone: 82-2-3010-4626

E-mail: jungbok.lee@gmail.com

Table S1. List of included studies and their endpoints

| NCT number | No of patients | Trough FEV_1_ | Change of trough FEV_1_ | FEV_1_ AUC 0-12h | FEV_1_ AUC 0-6h | FEV_1_ AUC 0-3h | % of patients TDI≥1 | SGRQ score | % of SGRQ responder | No. of acute exacerbation | No. of adverse events |
| --- | --- | --- | --- | --- | --- | --- | --- | --- | --- | --- | --- |
| NCT01727141 | 781 | o | o | o |  |  |  | o | o | o | o |
| NCT01712516 | 752 | o | o | o |  |  |  | o | o | o | o |
| NCT01682863 | 519 | o | o |  |  |  |  |  |  | o | o |
| NCT01777334 | 905 | o | o |  | o |  |  | o | o | o | o |
| NCT01316900 | 843 | o | o |  | o |  | o | o | o | o | o |
| NCT01313650 | 1252 | o | o |  | o |  |  | o | o | o | o |
| NCT01316913 | 869 | o | o |  | o |  | o | o | o | o | o |
| NCT02296138 | 7903 | o | o |  |  |  |  |  |  | o | o |
| NCT01964352 | 610 | o | o |  |  | o |  | o | o |  | o |
| NCT02006732 | 607 | o | o |  |  | o |  | o | o |  | o |
| NCT01431274 | 2624 | o | o |  |  | o |  | o | o | o | o |
| NCT01431287 | 2539 | o | o |  |  | o |  | o | o | o | o |

AUC, area under the curve; FEV_1_, forced expiratory volume at 1 s; NCT, National Clinical Trial; SGRQ, St. George’s Respiratory Questionnaire; TDI, transition dyspnea index.

Table S2. Baseline patient characteristics (excluding placebo arms) included in the 12 studies

| NCT number | Intervention | No. of patients | Age, years | Male | BMI, kg/m^2^ | FEV_1_, L | FEV_1_,  %pred. | Current smoker | F/U period |
| --- | --- | --- | --- | --- | --- | --- | --- | --- | --- |
| NCT01727141 | GLY/IND  GLY  IND | 260  261  260 | 64.3 ± 10.8  63.7 ± 10.3  63.9 ± 10.2 | 170 (65.4)  183 (70.1)  186 (71.5) | 27.2 ± 4.9  27.7 ± 5.2  27.6 ± 5.2 | 1.3 ± 0.4  1.3 ± 0.5  1.3 ± 0.5 | 46.1 ± 13.1  44.9 ± 13.4  45.2 ± 13.3 | 126 (48.5)  131 (50.2)  130 (50.0) | 12 w |
| NCT01712516 | GLY/IND  GLY  IND | 250  251  251 | 63.6 ± 9.2  63.6 ± 8.8  63.1 ± 9.2 | 1154 (61.6)  144 (57.4)  150 (59.8) | 27.2 ± 4.9  27.6 ± 5.7  27.7 ± 5.2 | 1.3 ± 0.5  1.3 ± 0.5  1.3 ± 0.5 | 45.0 ± 12.6  45.7 ± 13.3  45.7 ± 13.2 | 132 52.8  137 (54.6)  136 (54.2) | 12 w |
| NCT01682863 | GLY/IND  IND | 347  172 | 64.7 ± 8.1  63.1 ± 9.0 | 220 (63.6)  127 (73.8) | –  – | 1.2 ± 0.5  1.3 ± 0.5 | 45.1 ± 12.7  43.8 ± 11.7 | 167 (48.3)  85 (49.4) | 56 w |
| NCT01777334 | UMEC/VI  TIO | 454  451 | 61.9 ± 8.5  62.7 ± 8.5 | 310 (68.3)  303 (67.2) | 27.9 ± 5.9  26.8 ± 5.5 | 1.3 ± 0.5  1.3 ± 0.5 | 41.5 ± 12.6  41.5 ± 12.6 | 270(59.5)  243 (53.9) | 24 w |
| NCT01316900 | UMEC/VI  TIO  VI | 426  208  209 | 63.0 ± 8.8  62.6 ± 9.4  63.2 ± 9.1 | 299 (70.2)  140 (67.3)  143 (68.4) | 26.9 ± 5.7  27.6 ± 5.5  27.4 ± 5.7 | 1.3 ± 0.5  1.3 ± 0.5  1.3 ± 0.5 | 43.3 ± 13.4  43.8 ± 13.6  43.7 ± 13.3 | 222 (52.1)  99 (47.6)  106 (50.7) | 24 w |
| NCT01313650 | UMEC/VI  UMEC  VI | 413  418  421 | 63.1 ± 8.7  63.9 ± 9.1  62.7 ± 8.5 | 305 (73.9)  298 (71.3)  285 (67.7) | 27.3 ± 6.0  26.5 ± 5.6  26.6 ± 5.9 | 1.3 ± 0.5  1.2 ± 0.5  1.2 ± 0.5 | 42.8 ± 13.3  41.9 ± 13.6  42.6 ± 13.4 | 203 (49.2)  207 (49.5)  199 (47.3) | 24 w |
| NCT01316913 | UMEC/VI  UMEC  TIO | 432  222  215 | 64.4 ± 8.6  64.5 ± 8.3  65.2 ± 8.3 | 288 (66.7)  148 (66.7)  153 (71.2) | 26.6 ± 5.9  26.5 ± 5.7  26.4 ± 6.1 | 1.2 ± 0.5  1.1 ± 0.5  1.2 ± 0.4 | 42.0 ± 13.5  40.7 ± 12.7  42.0 ± 13.5 | 188 (43.5)  98 (44.1)  102 (47.4) | 24 w |
| NCT02296138 | TIO/OLO  TIO | 3951  3952 | 66.5 ± 8.4  66.3 ± 8.5 | 2793 (70.7)  2848 (72.1) | 26.3 ± 5.9  26.2 ± 5.8 | 1.2 ± 0.4  1.2 ± 0.4 | 46.4 ± 17.1  45.7 ± 11.9 | 1443 (36.5)  1486 (37.6) | 52 w |
| NCT01964352 | TIO/OLO  TIO | 406  204 | 64.7 ± 8.5  64.9 ± 8.1 | 230 (56.7)  125 (61.3) | 27.2 ± 5.6  28.3 ± 6.0 | 1.3 ± 0.5  1.3 ± 0.5 | 48.4 ± 13.6  47.8 ± 13.2 | 210 (51.7)  98 (48.0) | 12 w |
| NCT02006732 | TIO/OLO  TIO | 404  203 | 64.7 ± 8.5  64.7 ± 8.4 | 259 (64.1)  130 (64.0) | 28.1 ± 5.8  28.4 ± 6.4 | 1.4 ± 0.5  1.4 ± 0.5 | 47.9 ± 13.6  49.4 ± 12.9 | 182 (45.1)  91 (44.8) | 12 w |
| NCT01431274 | TIO/OLO  TIO  OLO | 1044  1052  528 | 64.5 ± 8.1  64.2 ± 8.5  63.7 ± 8.0 | 773 (74.0)  775 (73.7)  386 (73.1) | 25.6 ± 5.4  26.0 ± 5.5  25.6 ± 5.7 | 1.2 ± 0.5  1.2 ± 0.5  1.2 ± 0.5 | 43.7 ± 14.8  44.1 ± 15.1  43.7 ± 15.5 | 385 (36.9)  403 (38.3)  196 (37.1) | 52 w |
| NCT01431287 | TIO/OLO  TIO  OLO | 1015  1014  510 | 63.4 ± 8.0  63.7 ± 8.6  64.7 ± 8.3 | 717 (70.6)  734 (72.4)  378 (74.1) | 26.0 ± 5.7  25.8 ± 5.3  26.1 ± 5.8 | 1.2 ± 0.5  1.2 ± 0.5  1.2 ± 0.5 | 43.4 ± 15.1  43.3 ± 15.4  44.4 ± 15.1 | 387 (38.1)  355 (35.0)  182 (35.7) | 52 w |

Data are presented as mean ± SD or number (%). The number of patients are presented based on the full analysis set.

%pred., % of the predicted value; BMI, body mass index; FEV_1_, forced expiratory volume at 1 s; F/U, follow-up; GLY; glycopyrronium; IND, indacaterol; NCT, National Clinical Trial; OLO, olodaterol; SD, standard deviation; TIO, tiotropium; UMEC, umeclidinium; VI, vilanterol

Table S3. Criteria used to classify symptom severity in each study

| NCT number | Criteria for symptom severity |
| --- | --- |
| NCT01727141 | mMRC grade |
| NCT01712516 | mMRC grade |
| NCT01682863 | mMRC grade |
| NCT01777334 | mMRC grade |
| NCT01316900 | mMRC grade |
| NCT01313650 | mMRC grade |
| NCT01316913 | mMRC grade |
| NCT02296138 | CAT score |
| NCT01964352 | SGRQ score |
| NCT02006732 | SGRQ score |
| NCT01431274 | SGRQ score |
| NCT01431287 | SGRQ score |

CAT, COPD assessment test; mMRC, modified Medical Research Council; NCT, National Clinical Trial; SGRQ, St. George’s Respiratory Questionnaire.

Table S4. Changes in the trough FEV_1_ (L) according to the treatment arm in each study and treatment difference between dual and monotherapy.

| NCT number | LABA | | LAMA | | Dual | | Dual–LABA | | Dual–LAMA | |
| --- | --- | --- | --- | --- | --- | --- | --- | --- | --- | --- |
|  | mean | SD | mean | SD | mean | SD | Difference | 95% CI | Difference | 95% CI |
| NCT01727141 | 0.051 | 0.243 | 0.051 | 0.236 | 0.149 | 0.249 | 0.099 | 0.057–0.141 | 0.099 | -0.057–0.141 |
| NCT01712516 | 0.072 | 0.231 | 0.098 | 0.241 | 0.164 | 0.228 | 0.092 | 0.051–0.133 | 0.066 | 0.025–0.107 |
| NCT01682863 | 0.090 | 0.251 |  |  | 0.184 | 0.217 | 0.094 | 0.052–0.136 |  |  |
| NCT01777334 |  |  | 0.097 | 0.235 | 0.205 | 0.235 |  |  | 0.108 | 0.075–0.141 |
| NCT01316900 | 0.121 | 0.261 | 0.127 | 0.272 | 0.210 | 0.251 | 0.090 | 0.042–0.137 | 0.084 | 0.037–0.131 |
| NCT01313650 | 0.082 | 0.233 | 0.123 | 0.225 | 0.164 | 0.246 | 0.082 | 0.046–0.118 | 0.041 | 0.005–0.077 |
| NCT01316913 |  |  | 0.169 | 0.256 | 0.212 | 0.227 |  |  | 0.043 | 0.006–0.080 |
| NCT02296138 |  |  | -0.024 | 0.247 | 0.017 | 0.234 |  |  | 0.041 | -0.001–0.084 |
| NCT01964352 |  |  | 0.106 | 0.165 | 0.136 | 0.205 |  |  | 0.030 | -0.004–0.063 |
| NCT02006732 |  |  | 0.084 | 0.173 | 0.123 | 0.204 |  |  | 0.039 | 0.005–0.072 |
| NCT01431274 | 0.035 | 0.180 | 0.048 | 0.196 | 0.106 | 0.193 | 0.071 | 0.050–0.092 | 0.058 | 0.041–0.075 |
| NCT01431287 | 0.033 | 0.196 | 0.059 | 0.202 | 0.116 | 0.213 | 0.083 | 0.059–0.106 | 0.057 | 0.038–0.076 |
| Total |  |  |  |  |  |  | 0.087 | 0.068–0.097 | 0.061 | 0.049–0.072 |
| p-value  (overall effect) |  |  |  |  |  |  | <.0001 |  | <.0001 |  |

CI, confidence interval; FEV_1_, forced expiratory volume at 1 s; LABA, long-acting beta-2 agonist; LAMA, long-acting muscarinic antagonist; NCT, National Clinical Trial; SD, standard deviation.

Table S5. Rates of acute exacerbation per 6 months in patients treated with dual or monotherapy of LABA and/or LAMA

|  | Intervention | Mean rate of exacerbation per 6 months (SD) | Treatment difference vs. dual (95% CI) | p-value |
| --- | --- | --- | --- | --- |
| GOLD III/IV | Dual | 0.176 (0.275) |  |  |
|  | LABA | 0.212 (0.211) | 0.035 (-0.012–0.083) | 0.194 |
|  | LAMA | 0.188 (0.298) | 0.012 (-0.018–0.042) | 0.625 |
| GOLD I/II | Dual | 0.108 (0.395) |  |  |
|  | LABA | 0.139 (0.382) | 0.032 (-0.010–0.074) | 0.176 |
|  | LAMA | 0.122 (0.427) | 0.014 (-0.013–0.041) | 0.459 |

CI, confidence interval; GOLD, Global initiative for Chronic Obstructive Lung Disease; LABA, long-acting beta-2 agonist; LAMA, long-acting muscarinic antagonist; SD, standard deviation.

Figure S1. Study flow diagram


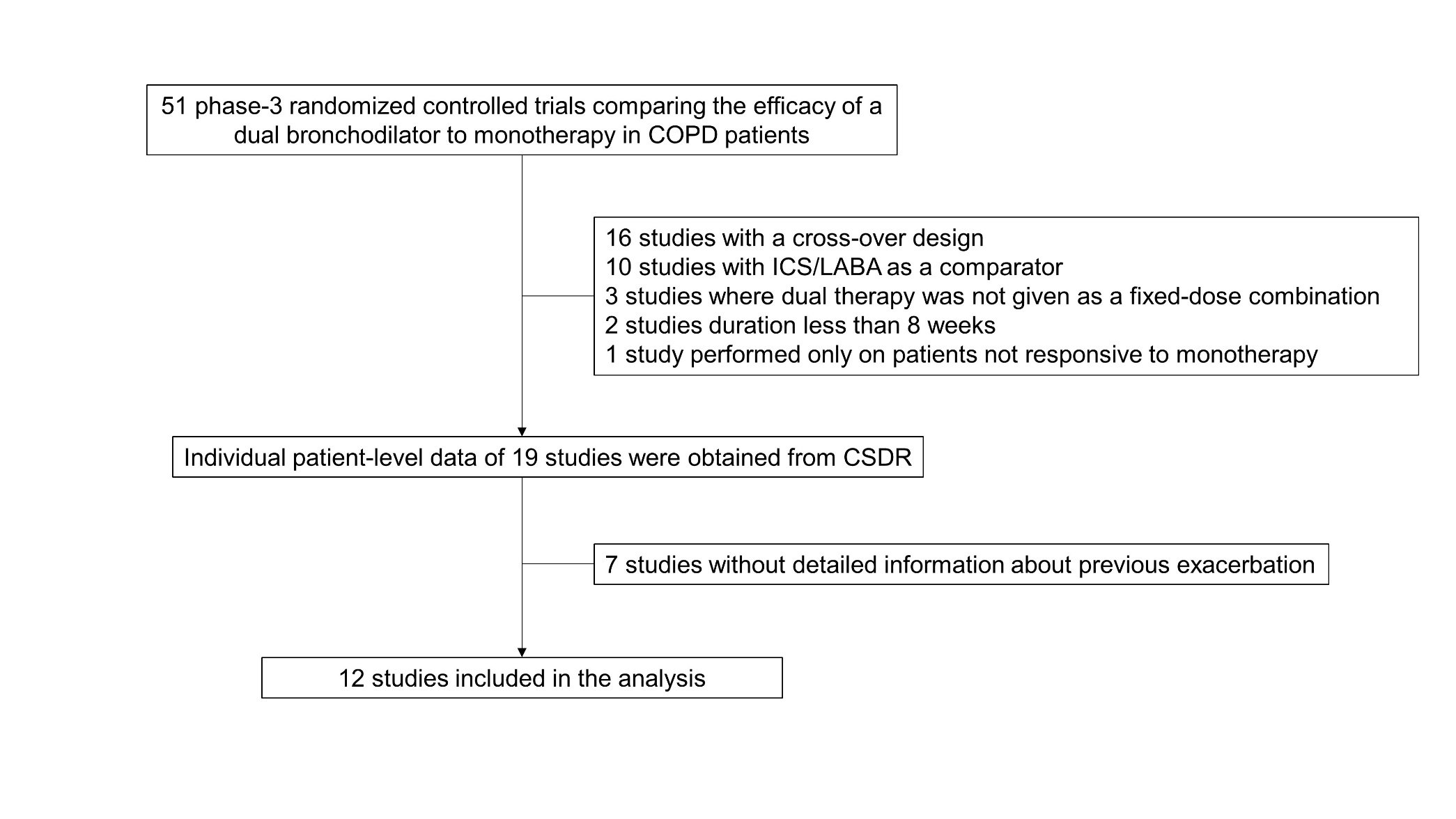


COPD, chronic obstructive pulmonary disease; CSDR, clinicalstudydatarequest.com; ICS, inhaled corticosteroid; LABA, long-acting beta-2 agonist.
